# Guidelines: The Do’s, Don’ts and Don’t Knows of Creating Open Educational Resources

**DOI:** 10.5334/pme.817

**Published:** 2023-01-09

**Authors:** Faran Khalid, Michael Wu, Daniel K. Ting, Brent Thoma, Mary R. C. Haas, Michael J. Brenner, Yusuf Yilmaz, Young-Min Kim, Teresa M. Chan

**Affiliations:** 1 Michael G. DeGroote School of Medicine, McMaster University, Hamilton, Ontario, Canada; 2 Department of Emergency Medicine, University of British Columbia, CA; 3 Department of Emergency Medicine, University of Saskatchewan, CA; 4 Department of Emergency Medicine University of Michigan Medical School, US; 5 Department of Otolaryngology — Head and Neck Surgery University of Michigan Medical School, US; 6 McMaster University Faculty of Health Sciences McMaster Education Research, Innovation and Theory (MERIT) program & Office of Continuing Professional Development Hamilton, Ontario, Canada; 7 Department of Medical Education, Faculty of Medicine, Ege University, Izmir, Turkey; 8 Department of Emergency Medicine, College of Medicine, The Catholic University of Korea, Seoul, South Korea; 9 McMaster University, Faculty of Health Sciences, Dept of Medicine, Division of Emergency, CA; 10 McMaster University, Faculty of Health Sciences, Office of Continuing Professional Development, Hamilton, Ontario, Canada

## Abstract

**Background:**

In medical education, there is a growing global demand for Open Educational Resources (OERs). However, OER creators are challenged by a lack of uniform standards. In this guideline, the authors curated the literature on how to produce OERs for medical education with practical guidance on the Do’s, Don’ts and Don’t Knows for OER creation in order to improve the impact and quality of OERs in medical education.

**Methods:**

We conducted a rapid literature review by searching OVID MEDLINE, EMBASE, and Cochrane Central database using keywords “open educational resources” and “OER”. The search was supplemented by hand searching the identified articles’ references. We organized included articles by theme and extracted relevant content. Lastly, we developed recommendations via an iterative process of peer review and discussion: evidence-based best practices were designated Do’s and Don’ts while gaps were designated Don’t Knows. We used a consensus process to quantify evidentiary strength.

**Results:**

The authors performed full text analysis of 81 eligible studies. A total of 15 Do’s, Don’t, and Don’t Knows guidelines were compiled and presented alongside relevant evidence about OERs.

**Discussion:**

OERs can add value for medical educators and their learners, both as tools for expanding teaching opportunities and for promoting medical education scholarship. This summary should guide OER creators in producing high-quality resources and pursuing future research where best practices are lacking.

## Introduction

The rapid expansion of remote and electronic learning has coincided with a digital transformation of the educational landscape, affecting how content is disseminated, applied, and integrated into practice. Open Educational Resources (OERs), defined as “teaching, learning and research materials in any medium, digital or otherwise, that reside in the public domain or have been released under an open license that permits no-cost access, use, adaptation and redistribution by others with no or limited restrictions” [1] are increasingly being used at all levels of medical education. They include freely accessible, openly licensed text, media, and other digital assets for teaching, learning, assessing, and research that may be re-mixed, improved and redistributed under some licenses [2].

Unfortunately, as podcasts, blogs, online journal clubs, online textbooks, and other forms of OER have proliferated, standards for quality have lagged, with potential negative impacts for the quality of the education provided via these newer media [3,4]. This is particularly relevant at a time when these resources must distinguish themselves from the substantial amount of medical misinformation being published online [5].

Evidence-based guidance in the creation of OERs would support increased quality and consistency in these resources while also ensuring alignment between resource design and learner needs [6,7,8]. We enlisted medical education experts to curate the available evidence with the goal of defining best practices in the creation of OERs in medical education.

## Methods

Development of this guideline proceeded in four stages: literature review; guideline development; consensus development; and grading. The literature review was conducted using a combination of the results from a rapid review of OER evaluation methods by Ting et al., a hand search of all references from the articles from the Ting review, and expert feedback from our guidelines’ senior authors who are well-published in this area [4]. These stages resulted in a comprehensive list of guidelines in the form of “Do’s, Don’ts, and Don’t Knows”.

### Authorship Team

We recruited leading medical educators from Canada, the United States, Turkey, and Korea to contribute. We identified expert participants by seeking individuals’ relevant expertise in medical education, open access resources, and previous experience in the development of guidelines or consensus documents from within a closed online research community of practice called the *Technology Education and Collaboration Hub* [9]. Additional authors were recruited due to their roles as frontline users of OERs (e.g. students, frontline teachers). We aimed for multidisciplinary representation, with representatives from medical specialties, surgical specialties, and the learning sciences.

### Literature Review

A articles for guideline development were aggregated from four sources. First, we procured all citations from the rapid review by Ting et al. which was a literature review on evaluation tools for OERs [4]. Second, we crowd-sourced additional studies from the authorship team relevant to OER creation. Third, we conducted a time-restricted search of OVID MEDLINE, EMBASE and Cochrane Central from 2010–2020 using the keywords “open educational resources” and “OER.” Fourth, we hand searched the reference lists of each included article for other relevant articles.

### Guideline Development

The lead authors (MW, FK, TMC) distilled key takeaway points and suggestions for OER curation and presented these findings for discussion with the larger authorship team. The authorship team provided input based on practical experience and literature. Through discussion and review, recommendations were developed and categorized. The lead authors then translated these recommendations into draft guidelines in the form of Do’s, Don’ts and Don’t Knows.

### Consensus Development

The guideline list and references were then circulated for all authors to suggest further evidence and edits. When the opinion was divided on classification of a guideline in discussion of Do’s and Don’ts, the guideline was moved into the Don’t Know category. After further refinement using comments provided by the authorship team, the lead authors circulated the guidelines for final approval.

### Grading

Guidelines were then graded for strength of evidence using the *Perspectives on Medical Education (PMED)* grading framework [10,11] by the lead authors (MW, FK, TMC).

## Results

**Figure 1:**
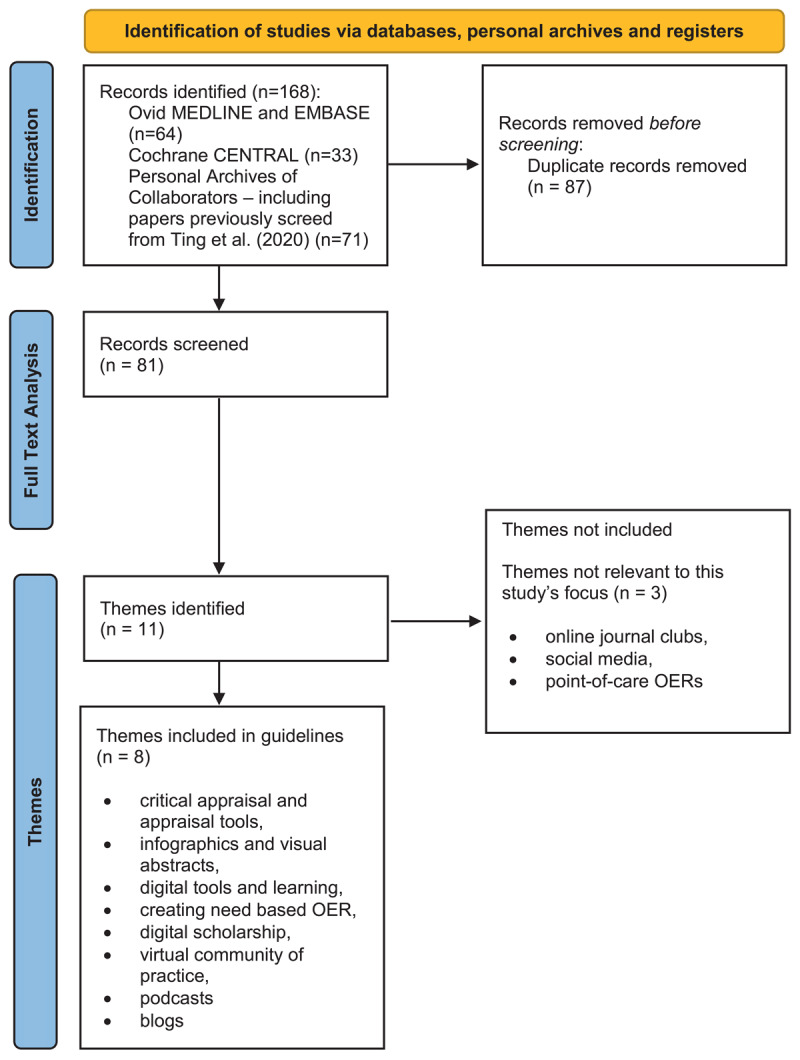
Flow diagram for included studies. Legend: OER = Open Educational Resource.

Eighty-one studies were initially included for full-text analysis and identification of key categories. The primary authors identified the key takeaways from these articles and formulated them as guidelines. Based on our thematic analysis of these studies, we developed 15 guidelines through a process of discussion and consensus-building. Figure 1 depicts our workflow. The studies that were ultimately included can be found in the reference section of our paper, while Appendix A shows all studies considered regardless of their inclusion in the final publication. After careful consideration and discussion within the group, only categories pertinent to the creation of OERs directed guideline development. A final list of 15 guidelines were derived from this process.

The background evidence for each guideline is described within each subsection. The strength of the recommendations is summarized in Table 1. The raw results table can be found in Appendix A.

**Table 1:** Consensus criteria for strength of recommendation.


GUIDELINE NUMBER	CLASSIFICATION OF DO’S, DON’TS AND DON’T KNOWS (RECOMMENDATION LEVEL IN BRACKETS)

1	Do: Conduct a needs assessment for OERs (Moderate)

2	Do: Create OER for rarely performed procedures or seldom encountered clinical presentations (Moderate)

3	Do: Use critical appraisal and tools to guide creation of OERs (Moderate)

4	**Do: Contribute to a virtual community of practice of educators interested in OER creation (Strong)**

5	Do: Use video archival to enhance online learning and education (Moderate)

6	Do: Teach learners to develop OERs as scholarship (Moderate)

7	**Do: Develop infographics and graphical abstracts by following criteria for content, formatting, and style (Strong)**

8	Do: Create and use podcasts as learning resources, knowing that trainees may be concurrently dual tasking (Moderate)

9	*Do: Consider creation of OERs that may be suitable for use at the point-of-care (Tentative)*

10	*Don’t: underestimate the uptake and influence of OERs in trainee education (Tentative)*

11	*Don’t: overlook the need to encourage learners to critically appraise OERs (even your own) (Tentative)*

12	*Don’t Know: Best practices for cross-language and cross-cultural sharing of OERs (Tentative)*

13	*Don’t Know: Efficacy of various types of OERs and best practices for OERs to optimize learning (Tentative)*

14	*Don’t Know: The ideal way to incorporate OERs into existing curricula (Tentative)*

15	*Don’t Know: Ethical considerations in use of OERs that are developed with industry support (Tentative)*


**Strong A large and consistent body of evidence.** Moderate Solid empirical evidence from one or more papers plus consensus of the authors. *Tentative Limited empirical evidence, but clear consensus of the authors*.

While most of the guidelines are applicable to all OERs, in some cases we focused our specific recommendations for particular types of OERs (e.g. infographics, blogs, and podcasts). For guidelines referencing specific types of OERs, the subtype is explicitly mentioned in the subheading.

### Guideline 1 – Do: Conduct a needs assessment for OERs

The complex process of OER creation often fails to consider the needs of the learners. Neglecting learner needs can result in irrelevant content ill-suited to address the curricular demands of its primary learners. Many studies, however, focus on understanding the content and the mode of delivery that learners desire [12,13,14,15]. The evidence from these papers should guide the creation of OER to better facilitate learning and fill in the gaps in OER. To understand learners’ needs, educators and content creators should conduct needs assessments through mechanisms such as surveys and focus groups to obtain student opinions. For example, Mallin et al. found that 80% of residents choose an OER topic for studying based on a recent clinical encounter [16], while Forestell and colleagues directly surveyed both trainees and educators to determine their curricular plan [12]. Studies like these can then help creators decide on topics for their OERs.

Assessing learner preference for medium can increase the likelihood that learners access and share content. For example, Mallin et al. found that emergency medicine (EM) residents prefer to listen to podcasts and social media instead of completing home reading assignments [16]. Given how many EM residencies operate on a rotating curriculum that covers all areas of their field, educators may consider suggesting timely resources like podcasts covering topics likely to be encountered by the resident in the near future. Although tailoring to learning styles has little evidence of effectiveness, adopting preferred formats can improve exposure [17]. Furthermore, an emphasis on active over passive format enhances learner engagement [18], although one study found that this is rare [19].

From the junior learner (medical student or first year resident) perspective, existing OERs often lack or underemphasize foundational content [20,21]. Online survey-based needs assessments can elucidate differences in educator and student perceptions on topic importance [12,13,22,23,24]. In 2020, Forestell et al. conducted an online survey-based needs assessment on 32 EM topics and value was assigned to each by educators and students [12]. Of the 32 topics, the authors identified 23 topics mutually agreed by learners and educators to be important for future OERs. This needs assessment identified general consistency between topics that students and educators deemed as high priority. However, EM educators valued some topics (i.e. chest pain, dyspnea) more highly than students, suggesting that students may not always self-identify their own knowledge gaps. Consequently, OER creators must seek out both the perspectives of learners and teachers to best identify the existing gaps in knowledge and/or curricula.

### Guideline 2 – Do: Create OERs for rarely performed procedures or seldom encountered clinical presentations

Educators should create more OERs for certain procedures and rare presentations that learners, including rural physicians, encounter less frequently during clinical care. Current literature often provides an abundance of content related to common conditions and a relative paucity of credible research related to more rare topics or procedures [23,25]. Expanding OER geared toward rural physicians can better account for varying geographical contexts among learners. In 2016, Folkl et al. employed a survey to rural and urban Canadian-based EM physicians on their usage of EM resources and their self-reported confidence in EM domains [23]. The study identified that rural and urban emergency physicians perceive their own knowledge levels differently, particularly knowledge related to critical care. This difference in perceived knowledge may reflect less exposure by rural EM physicians to critically ill patients or the need for a wider knowledge base among ruralists who may lack access to specialists. As such, OERs must further address certain topics in greater detail to include rural learners. This observation should also apply to the context of other healthcare professional populations. We make an inference based on this needs assessment that OERs should likely also cover seldom encountered clinical presentations.

### Guideline 3 – Do: Use critical appraisal and tools to guide creation of OERs

As OERs have emerged as an important resource within medical education, several tools have been developed to evaluate their quality. Ting et al. (2020) published a rapid review that identified different quality assurance tools that were designed for these resources [4]. Table 2 provides an overview of the critical appraisal tools outlined in this review with the addition of the recently published revised Approved Instructional Resources (AIR) Score [26].

**Table 2:** Overview of critical appraisal tools outlined in this review.


TOOL	SUMMARY	SOURCE

Quality checklists for blogs and podcasts [101]	The quality checklists were the first of the tools published for medical education. They were derived from lists of quality indicators identified within Delphi studies. They could support both the review and creation of OERs.	Paterson QS, Colmers IN, Lin M, Thoma B, Chan T. The quality checklists for health professions blogs and podcasts. The Winnower. 2015;1–7. Available at: https://thewinnower.com/papers/2641-the-quality-checklists-for-medical-education-blogs-and-podcasts

rMETRIQ Score [70]	The METRIQ-5 and METRIQ-8 scores were derived from the same quality indicators as the quality checklists. The revised METRIQ (rMETRIQ) score is an optimized version that was clarified through a utilization study. It aims to provide a quality score that could be applied with little expertise [70].	Revised METRIQ score grading tool: https://metriqstudy.org/the-rmetriq-score-a-quality-assessment-tool-for-the-critical-appraisal-of-foam/

rAIR Score [26]	The ALiEM AIR Score was developed by emergency medicine educators to rate OERs for their learner. The revised AIR (rAIR) score was optimized and simplified through a utilization study but serves the same purpose [26].	Revised AIR score grading tool: https://europepmc.org/articles/PMC8194147/figure/aet210601-fig-0001/

MEWQET MEOW CCMEWQET	These three scores have a common origin. MEWQET, developed by pathology educators, was modified into MEOW by otolaryngology educators. These two tools informed the creation of CCMEWQET by critical care educators. They provide a specialty-specific lens to the evaluation of OERs.	MEWQET: https://www.jpathinformatics.org/articles/2013/4/1/images/JPatholInform_2013_4_1_29_120729_sm11.jpg MEOW: https://journalotohns.biomedcentral.com/articles/10.1186/s40463-017-0220-4/tables/1 CCMEWQET: https://journals.sagepub.com/na101/home/literatum/publisher/sage/journals/content/jica/2019/jica_34_1/0885066618759287/20181129/images/large/10.1177_0885066618759287-table1.jpeg


In the same way that quality evaluation tools inform the conduct of research, these quality evaluation tools could support OER developers in the development of high-quality resources. By ensuring that their resources meet the quality indicators of relevant tools, it is likely that creators will increase the quality of their work.

### Guideline 4 – Do: Contribute to a virtual community of practice of educators interested in OER creation

A community of practice (CoP) is formed when a group of professionals who share an interest engage in a social context conducive to participatory learning [27]. Although CoPs differ widely, three unifying attributes are mutual engagement, joint enterprise, and shared repertoire [27,28,29,30]. Mutual engagement arises from the chemistry of social relationships that bind members together, whereas joint enterprise refers to the set of goals that they share. Members cultivate a shared repertoire as they develop mutually understood knowledge, techniques, ideas, and terminology. A virtual CoP shares many of the same features of a regular CoP, except that the primary mode of communication between members occurs in the virtual space, using online communication tools [28,29,30]. In recent years, virtual CoPs have gained increasing traction, and a growing number have started to emerge around OERs [31,32].

There are several compelling reasons to join a virtual CoP. CoPs unite individuals who share a passion. Additionally, establishing blended networks of faculty and trainees affords a structure for facilitating meaningful coaching, mentorship, and sponsorship relationships [31,33]. For trainees, connecting with mentors can introduce them to optimizing social media use by avoiding pitfalls such as professionalism lapses and learning the basics of OER creation [32,34,35]. For educators, CoPs can galvanize collaborative scholarship, achieving a “critical mass” of motivated individuals with whom to share work and ideas [31,35]. Additionally, emerging evidence suggests that educators may use the virtual CoP in novel ways that facilitate high quality scholarship, such as to help recruit participants for research studies [36,37]. Trainees, on the other hand, can add further value to the CoP by providing bidirectional mentorship by sharing how they use modern technologies and programs which in turn can assist faculty in their creation of OERs.

For many blog-based OERs, submissions are welcomed from their online audience of trainees to consider for publication [38]. Some have introduced a modified peer-review process for submissions that seeks to both elevate the quality of the work and provide a positive academic experience through transparent coaching from faculty physicians [34,38]. To facilitate further progression from peripheral to core membership within the CoP, organizations have created formal training curricula through apprenticeships that pair trainees with experienced mentors in OER production [32,34,35,38,39].

### Guideline 5 – Do: Use video archival to enhance online learning and education

Educators should continue to use videoconferencing with archival (e.g. recording a Zoom webinar/talks and then sharing via a social video streaming service like YouTube or Vimeo) for open dissemination of knowledge and overcoming distance and access barriers, a need amplified by the physical distancing necessitated during the COVID-19 pandemic. Educators must invest in applying new technologies since they can help in: 1) improving project collaboration; 2) creating virtual meetings; 3) fostering digital mentorship; 4) forming virtual communities of practice; and 5) advancing online learning in the realms where remote e-work is unavoidable and where previously communication may have been hindered by a lack of in-person activities [40,41].

### Guideline 6 – Do: Teach learners to develop OERs as scholarship

In *Scholarship Reconsidered*, Boyer argued that a narrow view of scholarship as experimental research is incomplete and that an expanded definition that includes integration, application, and teaching is needed to capture the work of academics [42]. Boyer’s expanded framework for scholarship paved the way for enhanced legitimacy of OERs as the widespread adoption of the internet followed in the 1990s and 2000s [43]. Thoma et al. describe examples of digital products that parallel traditional scholarly output, largely falling under the “teaching and learning” category: interactive resources (i.e., online discussion boards, social networks and wikis), independent study resources (i.e., e-mail, online courses, serious games, virtual reality, web-based and computer-assisted learning), audiovisual resources (i.e., podcasts, video podcasts, and instructional videos), point-of-care resources (i.e., applications), written resources (i.e., online textbooks, blogs, open access journals and websites), and resource repositories (i.e., online repositories and search engines) [44]. Sherbino and colleagues later expanded this definition to specifically examine social media-based scholarship [45]. Husain et al. define digital scholarship as “original content that is disseminated digitally, whether that content is research, teaching materials, enduring resources, commentaries, or other scholarly work” [46].

Learners can be supported by faculty to develop OERs as scholarship through formal curricular teaching methods. Murray et al. studied the effectiveness of teaching evidence-based medicine to medical students using Wikipedia [47]. First year medical students in small groups were tasked to choose a medical Wikipedia article to appraise and edit. Students excelled at identifying knowledge gaps and selecting appropriate literature for edits. Positioning medical learners as critical appraisers of existing digital resources actively engaged them in OER creation. However, students faced challenges including the technical aspect of editing Wikipedia and difficulty collaborating with the greater Wikipedia community. This highlights that although novel digital assignments and tools can facilitate medical learning, technical and social barriers accompany them.

### Guideline 7 – Do: Develop infographics and graphical abstracts by following criteria for content, formatting, and style

Infographics and graphical abstracts (including visual abstracts) are powerful tools that not only prompt individuals to take notice of content, but also serve to frame the overarching message and takeaways [48]. The salience of these visually oriented tools underscores the need to adhere to best practice in content, formatting, and style, with an eye toward minimizing potential sources of bias. Scientists and scholars have increasingly leveraged these visual tools [49,50,51,52], a trend that parallels the overall accelerating adoption of technology in medical education [53]. Graphical abstracts are a relatively new entrant to the educational and scientific community. While no uniformly agreed-on standards exist, several styles have emerged.

The *visual abstract style*, introduced in 2016, is arguably most relevant to OER creation [49]. Visual abstract style infographics are used primarily by scholarly journals and consist of a title and key findings (text and visual icon), showcasing the most important data [52]. Visual abstracts are usually created with digital software and can increase engagement by healthcare professionals, particularly on social media [54]. The *infographic style* is less specific to medical journals and often professionally produced using specialized software; the intended audience may be academicians [55,56,57] or the general public [58]. The *diagram style* was developed decades prior for use in specialized fields [50]. Last, the *comic style* conveys research findings, combining humorous illustration with minimal text [59]. Among these, the *visual abstract* is most relevant to medical educators, as *visual abstracts* are straightforward to develop, useful for scholarly endeavors, and increasingly used on social networking platforms [49].

The *visual abstract* needs to accurately translate the written abstract without distortion, and therefore the need for standardization is more stringent than for other infographics. Visual abstracts should give context to the study; explicitly state the quality of the evidence; and minimize reporting bias if applicable. To maximize engagement, the layout of abstracts should be clear and organized with easy-to-read fonts and high-resolution images. Also, these images should be reproducible in grayscale. The source of data should be transparent, including author names, degrees, and full citation. Consumers often prefer visual abstracts as they cause less cognitive load, although visual abstracts do not necessarily improve delayed information retention [57].

Infographics and visual abstracts are alluring to readers due to imagery and succinctness, but this attribute also makes them inherently susceptible to misinterpretation. This is especially important as accelerated dissemination of research via social networks may improve uptake of an article, but this rapidity also makes it potentially challenging to rein in misconceptions [55,56].

The bias toward positive results — a tendency long documented in the scientific literature [60,61] — is of particular concern with visual abstracts. Care must be taken to avoid oversimplification [62], and overgeneralizations. Out of necessity, visual abstracts tend to be pared down, and often lack key statistical measures of uncertainty or inaccuracy, such as confidence intervals. Furthermore, visual abstracts are often created outside of the peer-review process. A few practices can help mitigate these risks of bias. For example, having both an internal and external review before posting on social media (e.g., Twitter) provides layers of quality control [63].

### Guideline 8 – Do: Create and use podcasts as learning resources, knowing that trainees may be concurrently dual tasking

Students and educators increasingly utilize podcasts [64]. Podcasts can also effectively disseminate information and learning objectives across various regional learning institutions.

Podcasts are easy to use and engaging, enabling both broad exposure to content and targeted learning [7]. Being able to multitask, listening to podcasts while doing other activities is a unique advantage of podcasts. The learners can use their time productively [7,65]. A recent randomized controlled trial showed that listening to podcasts while driving did not significantly decrease retention both 30-minutes immediately after (initial recall) and 30-days later (delayed recall) compared to undistracted listening [66].

Educators should examine usage patterns of podcasts to help guide specifics of content development such as length of an episode. Most listeners used podcasts for less than 30 minutes [65,67] and less than 2 hours per week [68]. Educators should also be aware of what motivates learners to use podcasts (e.g., learning content and staying up to date were main motivators) [65,67,68].

Based on the current evidence, podcasts should be less than 30-minutes and deal with up-to-date information. Podcasts generally do not incorporate active learning and thus necessitate complementary resources. Gestalt ratings from approximately 20 health professionals are required to reliably assess podcast quality [17], so consulting broadly and getting wide opinions may be worthwhile prior to OER release.

### Guideline 9 – Do: Consider creation of OERs that may be suitable for use at the point-of-care

Educators and students alike can utilize them to answer clinical questions in real-time that enhance patient care and education. OERs can thus serve as a valuable “point of care” (POC) resource, which is defined as “any reference material used in the provision of medical care directly at the bedside and may include clinical problem-solving, patient care, patient education, or learner education” [15]. When creating POC resources, a study by Patocka et al. generated a conceptual framework for how EM providers use POC resources that describes four main purposes: deep-dive, advanced clinical decision making, teaching patients and teaching learners [15]. Junior learners tend to prioritize increasing their depth of knowledge (“deep dive”), whereas more senior learners seek to answer specific clinical questions through small bursts of knowledge-seeking prompted by scenarios [15]. Additionally, senior learners and practicing physicians tend to use POC not only for themselves but to disseminate knowledge to others, including both patients and learners, thereby freeing up time for other tasks [15]. OER creators can be informed by this literature to better tailor the resource to the use and user.

### Guideline 10 – Don’t: underestimate the uptake and influence of OERs in trainee education

This recent expansion of asynchronous OERs has spurred a movement away from traditional textbooks and synchronous classroom-based activities, particularly among more recent generations of learners. As early as 2015, a survey of Canadian EM residents found that over 90% of respondents had used OERs for general EM education, procedural skills, and diagnostic test interpretation, with the most commonly used resources including wikis, file-sharing websites, textbooks, and podcasts [6]. A 2014 survey of American EM residents found that roughly 98% engaged in at least an hour of educational activities outside of traditional residency curricula, with listening to podcasts more commonly reported than time reading textbooks [16]. Most residents perceived podcasts to have the greatest benefit, over other resources such as textbooks, journals, and Google [6]. The global uptake of asynchronous OERs has occurred, although more slowly. A 2013 global survey of 44 trainees demonstrated that 82% were aware of blogs, 80% of websites, 75% of podcasts and 61% of Twitter as EM educational resources, with trainees in lower income settings generally less aware of specific resources despite lack of internet access not appearing to be a major barrier to use [69]. This, however, is not universal across all modalities – podcasts were noted to have less uptake in low- and middle-income contexts [24].

### Guideline 11 – Don’t: overlook the need to encourage learners to critically appraise OERs (even your own)

Although asynchronous OERs come with many advantages, Mallin et al. identified a concerning finding: trainees utilizing asynchronous OERs reported rarely evaluating the primary sources or the quality of the evidence [16]. Again, generational differences have been noted, with program directors more likely than residents to access primary references [6]. While the use of online educational resources among trainees is inevitable, these findings highlight the need for educators to teach them about critical appraisal including quality indicators, such as the revised METRIQ score or the AIR score [26,70,71]. Some literature also suggests it may also be worthwhile to explore new ways of teaching critical appraisal skills of these OERs alongside the original peer-reviewed papers [72]. While many have written about the role that OER producers must play in encouraging active critical appraisal of the peer-reviewed literature [73,74,75], some literature reminds us that the critical appraisal of OERs themselves must also be incorporated into the readers’ skills. This skill should be encouraged by educators, but also advocated by those who serve as OER creators, curators, or editors [4,8,72,76].

### Guideline 12 – Don’t Know: Best practices for cross-language and cross-cultural sharing of OERs

The one key advantage of OER is the easy dissemination and accessibility of resources across the globe. However, the paucity of multilingual OER repositories represents a barrier to access. Moreover, the differing contexts and cultures of health globally may necessitate more than simple language translation to achieve the same relevance of a given OER to different audiences. Although OERs allow for creation of content that appeals to learners in many different countries that speak many different languages, it remains unclear how to best improve accessibility of content and improve cross-language sharing of resources [3,24]. It is also unclear how OERs fare in the varying cross-cultural contexts and whether they reinforce a certain way of thinking or introduce biases. While OERs are often used in the Global South [77], we must be mindful that in other medical education systems there have been barriers to engagement [78], and attending to these barriers in OERs will be a persistent challenge to overcome.

### Guideline 13 – Don’t Know: Efficacy of various types of OERs and best practices for OERs to optimize learning

While there is some evidence that OERs of different formats (e.g., blogs vs podcasts) can have similar learning outcomes [65,79,80,81], additional research must clarify best practices for learning optimization. Some research has been completed to understand what listeners feel make podcasts most effective [17,18,67]. For instance, a recent study has shown that interpolated questions within a podcast may improve knowledge retention [18]. However, additional empirical studies will need to further clarify which attributes enhance learner experience and outcomes. Interestingly, research can sometimes show counterintuitive findings. Several studies have shown that participants often dual-task while listening to podcasts [17,65,67,82] however, at least one randomized controlled trial has shown that dual-tasking with a driving simulator has little effect on learning outcomes [66].

### Guideline 14 – Don’t Know: The ideal way to incorporate OERs into existing curricula

The approach that learners exhibit when digesting OER content, such as podcasts, often deviates from traditional active learning behaviors like notetaking and repeating [17]. It is still unclear as to how OERs might be best harnessed to augment, complement, or replace traditional learning activities. As such, there remains an uncertainty on how medical educators can best integrate OERs into traditional medical curricula. Traditional learning is done in environments where more active learning behaviors such as note taking can be exhibited, whereas OERs like podcasts are often consumed in situations like driving or exercise where such active learning behaviors are not easily observed.

While there is some promising evidence that OERs may be better than textbooks in some circumstances [81], this has not held true across all studies for all OER comparisons to traditional formats [83], suggesting that more nuanced research is required to understand when OERs may be most appropriate for learning. With increasing strain and burnout in faculty and trainee groups alike, it may be useful to engage in further research to determine what modalities might be equivalent to lectures or textbooks to decrease the burden of synchronous learning experiences. It is important to understand the place of traditional resources versus OERs in learning and investigate how to best integrate OERs into an educational strategy. These questions are important when considering needs based OER creation.

### Guideline 15 – Don’t Know: Ethical considerations in use of OERs that are developed with industry support

Partnerships between industry and academic centers have played an important role in bridging the gap between discovery and clinical implementation, but industry funding for development of such resources is fraught with potential of bias [84,85,86]. Whereas most refereed academic journals require disclosure of funding, such requirements are less standardized in OERs and are often absent [86]. The authors advocate for transparency around funding sources and other potential sources of bias in informational resources, but strategies to ensure this transparency are still evolving. Best practice, however, is for individuals creating such content to disclose financial or other potential conflicts that may have relevance to the information provided. When OERs involve industry support, unrestricted educational grants are preferable, as they minimize likelihood of sponsor influence on content. Educators should use due diligence in ensuring that material recommended to learners is free of commercial bias and made fully transparent.

## Discussion

**Figure 2:**
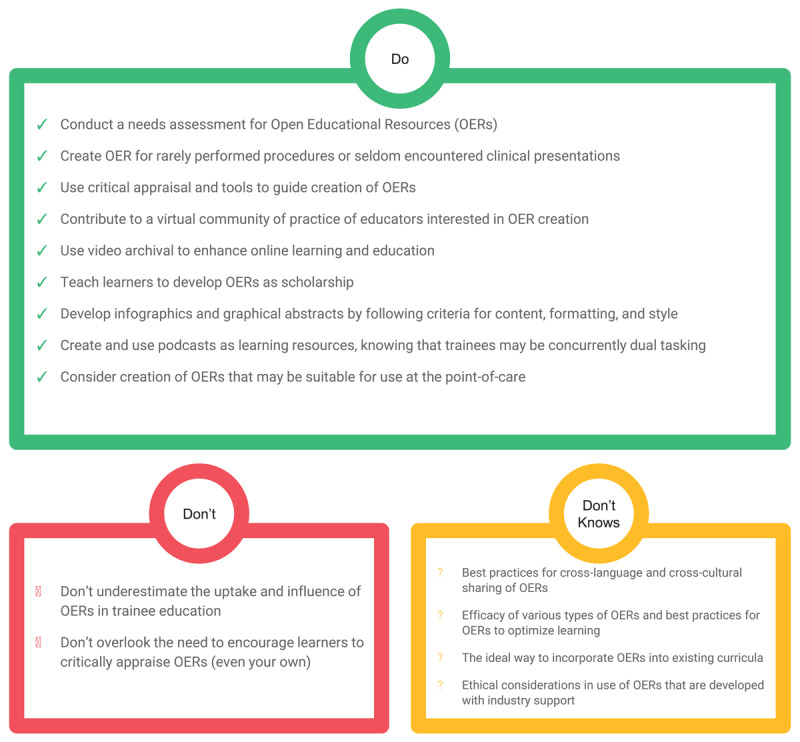
Summary graphic: Guidelines and background evidence for OER creation.

We have presented 15 guidelines (9 Do’s, 2 Don’ts, and 4 Don’t Knows) for scaffolding the creation of OERs within medical education based on a critical reading of the literature. There are still many unanswered questions within this burgeoning new area of scholarship, and we hope that the “Don’t Knows” inspire others from diverse backgrounds to ask key questions about how OERs might be incorporated into medical education. Please see Figure 2 for a summary of our findings.

As guidelines for the production of OERs are developed, these learning formats have become an increasingly legitimized form of educational scholarship. Sherbino et al. outlined four criteria for social media to be considered scholarship that could be adapted for these resources, concluding that it must: 1) reflect original content, 2) advance the field of health professions education by building on theory, research or best practice, 3) be archived and disseminated, and 4) provide the health professions education community with the ability to comment on and provide feedback in a transparent fashion that informs wider discussion [45]. Additionally, while not developed specifically for digital scholarship, Glassick’s criteria for evaluating scholarship provide a valuable starting point and include that scholarship must demonstrate clear goals, adequate preparation, appropriate methods, significant results, effective presentation and reflective critique [87]. Creators of OERs can use these criteria to scaffold their processes to ensure that these resources are rigorous.

There are ever-looming threats to the OER movement. A recent study showed that there may be a decline in the number of OER producers [88]. The sustainability of OER production may very well depend on how academics and/or readers begin to value their continued existence [89,90]. If OERs are considered scholarship it becomes important to quantify their value. Husain et al. provide recommendations for presenting digital scholarship to promotion and tenure committees that included demonstrating scholarship criteria, providing external evidence of impact through the use of some of the previously described metrics, including digital peer-review roles, citing digital scholarship consistently, crafting a digital scholarship mission statement, using traditional frameworks such as the teaching portfolio [46]. Cabrera et al. also provide recommendations for both scholars and academic institutions with regard to preparing and interpreting promotion packets that include digital scholarship [91]. The formal acknowledgement of OERs as scholarship could help producers to get academic credit towards promotion, which could also improve the sustainability of OER production.

Unfortunately, when it comes to evaluating the impact and quality of OERs, metrics applied to traditional scholarship such as journal impact factor and number of citations do not translate well to digital scholarship [91,92,93,94]. Alternative impact metrics for educational scholarship could include page views, time spent on a page, reactions (e.g., likes, dislikes, favorites), impressions, dissemination, unique users, geographic reach, followers on professional social media accounts, the Social Media Index [95,96], Alexa Ranking, and Altmetrics [46,97,98]. Alternative quality metrics have also been developed including the METRIQ score [70,99], the Social Media Index [95,96], Approved Instructional Resources (AIR) score [26,71,100], and the Quality Checklists for Health Professions Blogs and Podcasts [101]. These tools could be used by promotion and tenure committees to adapt their criteria [46,102] in a way that encompasses a wider view of what can be considered scholarship [103].

### Limitations

This guideline has several limitations to consider. Firstly, while this guideline has sought to aggregate the evidence regarding OER creation, this is certainly an evolving field. New articles will undoubtedly appear prior to publication of this guideline. Secondly, since our review focused on the guiding literature in medicine about OER creation, we may have missed guideline literature from surgical fields or other diagnostic fields. Thirdly, there is certainly a bias within the guideline towards EM citations and literature – and this may be due to a few different causes: 1) there is a preponderance of published literature by the EM community around OERs due to the naming of the Free Open Access Medical education movement at an EM conference but also the coincidental founding of a specialty-related journal; 2) several of the authors as well as published consulting experts identify as EM physicians and may have skewed our awareness of certain bodies of literature over others; 3) members of the EM community (including members of this authorship team) may preferentially use the term OER in their publications and this may have created a bias in our search findings. In future guidelines, dedicated searches of certain modalities such as blogs, podcasts, and specific social media platforms may be helpful to eliminate this bias.

## Conclusion

This article culminates key evidence into a guideline for the creation of OERs. We hope that the field will continue to evolve its practices by addressing the Don’t Knows, bringing further clarity to the Do’s and Don’ts of the field.
